# Genome Sequence of *Spizellomyces punctatus*

**DOI:** 10.1128/genomeA.00849-16

**Published:** 2016-08-18

**Authors:** Carsten Russ, B. Franz Lang, Zehua Chen, Sharvari Gujja, Terrance Shea, Qiandong Zeng, Sarah Young, Christina A. Cuomo, Chad Nusbaum

**Affiliations:** a Broad Institute of Harvard and MIT, Cambridge, Massachusetts, USA; b Robert Cedergren Centre for Bioinformatics and Genomics, Département de Biochimie, Université de Montréal, Montreal, Québec, Canada

## Abstract

*Spizellomyces punctatus* is a basally branching chytrid fungus that is found in the *Chytridiomycota* phylum. *Spizellomyces* species are common in soil and of importance in terrestrial ecosystems. Here, we report the genome sequence of *S. punctatus*, which will facilitate the study of this group of early diverging fungi.

## GENOME ANNOUNCEMENT

*Spizellomyces punctatus* is the type species of the fungal genus *Spizellomyces*, which is in the phylum *Chytridiomycota* (1). Like all chytrids, *Spizellomyces* produces uniflagellated zoospores during its reproductive cycle, but in contrast to other zoosporic fungi (e.g., *Allomyces*), spizellomycetalean zoospores can be ameboid while actively swimming, and the flagellar insertion site may move to a lateral position (2). *Spizellomyces* species are exclusively terrestrial (3). They are common in soil and of importance in terrestrial ecosystems—with both beneficial and detrimental impacts—found in association with a range of mycorrhizal fungi, mildews, plants, and soil nematodes (4, 5). In biochemical research, *S. punctatus* has gained attention because of the presence of mitochondrial 5′ tRNA editing (6), a form of post-transcriptional RNA processing previously only known from the unrelated ameboid protist *Acanthamoeba castellanii*. The *S. punctatus* genome was sequenced as part of the Origins of Multicellularity project; in addition to *S. punctatus*, this project sequenced genomes of several primitive eukaryotes to investigate commonalities and differences underlying multicellularity in animals and fungi (7).

To sequence the genome of *S. puncatus*, genomic DNA was extracted from strain DAOM BR117. For genome sequencing, we constructed three libraries, 4 and 10 kb plasmids and 40 kb Fosmids, and generated paired-end reads using Sanger chemistry. The reads were assembled using Arachne (8) Assemblez-Build 20090202 from roughly 11.7-fold sequence coverage; during manual review, gaps were newly introduced to address misassemblies or were closed based on read support.

Based on the assembly, the genome size was estimated to be 24.13 Mb with a G+C content of 47.6%. The assembly was organized in 329 contigs, which are linked by paired end reads into 38 scaffolds. The average base is found in a scaffold of *N*_50_ size 1.45 Mb and a contig of *N*_50_ size 155.89 kb. A total of 8,952 protein coding genes and 9,424 transcripts were predicted by combining the output from different annotation methods and RNA-Seq as previously described (9).

*S. punctatus* is the first member of the order *Spizellomycetales* to be sequenced and an important representative of *Chytridiomycota*, as one of only three species to have annotated genomes released in NCBI. This report is a major step in genomic studies of this basal group of fungi.

### Accession number(s).

The whole-genome sequence and annotation of *S. punctatus* isolate DAOM BR117 have been deposited at DDBJ/EMBL/GenBank under the accession number ACOE00000000. The version described in this paper is version ACOE01000000.

## References

[1] Hibbett DS, Binder M, Bischoff JF, Blackwell M, Cannon PF, Eriksson OE, Huhndorf S, James T, Kirk PM, Lücking R, Thorsten Lumbsch H, Lutzoni F, Matheny PB, McLaughlin DJ, Powell MJ, Redhead S, Schoch CL, Spatafora JW, Stalpers JA, Vilgalys R, Aime MC, Aptroot A, Bauer R, Begerow D, Benny GL, Castlebury LA, Crous PW, Dai YC, Gams W, Geiser DM, Griffith GW, Gueidan C, Hawksworth DL, Hestmark G, Hosaka K, Humber RA, Hyde KD, Ironside JE, Koljalg U, Kurtzman CP, Larsson KH, Lichtwardt R, Longcore J, Miadlikowska J, Miller A, Moncalvo JM, Mozley-Standridge S, Oberwinkler F, Parmasto E, Reeb V, Rogers JD, Roux C, Ryvarden L, Sampaio JP, Schussler A, Sugiyama J, Thorn RG, Tibell L, Untereiner WA, Walker C, Wang Z, Weir A, Weiss M, White MM, Winka K, Yao YJ, Zhang N 2007 A higher-level phylogenetic classification of the fungi. Mycol Res 111:509–547. doi:10.1016/j.mycres.2007.03.004. 17572334

[2] Barr DJS 1984 Cytological variation in zoospores of *Spizellomyces* (*Chytridiomycetes*). Can J Bot 62:1202–1208. doi:10.1139/b84-162.

[3] Barr DJS 1980 An outline for the reclassification of the *Chytridiales*, and for a new order, the *Spizellomycetales*. Can J Bot 58:2380–2394. doi:10.1139/b80-276.

[4] Lozupone CA, Klein DA 2002 Molecular and cultural assessment of chytrid and *Spizellomyces* populations in grassland soils. Mycologia 94:411–420. doi:10.2307/3761775. 21156512

[5] Paulitz TC, Menge JA 1984 Is *Spizellomyces punctatum* a parasite or saprophyte of vesicular-arbuscular mycorrhizal fungi? Mycologia 76:99–107. doi:10.2307/3792840.

[6] Laforest MJ, Roewer I, Lang BF 1997 Mitochondrial tRNAs in the lower fungus *Spizellomyces punctatus*: tRNA editing and UAG “stop” codons recognized as leucine. Nucleic Acids Res 25:626–632. doi:10.1093/nar/25.3.626. 9016605 PMC146481

[7] Ruiz-Trillo I, Burger G, Holland PW, King N, Lang BF, Roger AJ, Gray MW 2007 The origins of multicellularity: a multi-taxon genome initiative. Trends Genet 23:113–118. doi:10.1016/j.tig.2007.01.005. 17275133

[8] Jaffe DB, Butler J, Gnerre S, Mauceli E, Lindblad-Toh K, Mesirov JP, Zody MC, Lander ES 2003 Whole-genome sequence assembly for mammalian genomes: Arachne 2. Genome Res 13:91–96. doi:10.1101/gr.828403. 12529310 PMC430950

[9] Haas BJ, Zeng Q, Pearson MD, Cuomo CA, Wortman JR 2011 Approaches to fungal genome annotation. J Mycol 2:118–141. 10.1080/21501203.2011.606851 PMC3207268 22059117

